# Metal Oxide Thin-Film Heterojunctions for Photovoltaic Applications

**DOI:** 10.3390/ma11122593

**Published:** 2018-12-19

**Authors:** Ørnulf Nordseth, Raj Kumar, Kristin Bergum, Laurentiu Fara, Constantin Dumitru, Dan Craciunescu, Florin Dragan, Irinela Chilibon, Edouard Monakhov, Sean Erik Foss, Bengt Gunnar Svensson

**Affiliations:** 1 Institute for Energy Technology (IFE), P.O. Box 40, NO-2027 Kjeller, Norway; Sean.Foss@ife.no; 2 Department of Physics/Center for Materials Science and Nanotechnology (SMN), University of Oslo, P.O. Box 1048, Blindern, NO-0316 Oslo, Norway; raj.kumar@smn.uio.no (R.K.); kristin.bergum@smn.uio.no (K.B.); edouard.monakhov@fys.uio.no (E.M.); b.g.svensson@fys.uio.no (B.G.S.); 3 Department of Physics, Faculty of Applied Sciences, Polytechnic University of Bucharest, Spl. Independentei 313, RO-060042 Bucharest, Romania; lfara@renerg.pub.ro (L.F.); condumitru@yahoo.com (C.D.); dan.craciunescu@sdettib.pub.ro (D.C.); florin.dragan@sdettib.pub.ro (F.D.); 4 Academy of Romanian Scientists, Spl. Independentei 54, RO-030167 Bucharest, Romania; 5 National Institute of Research and Development for Optoelectronics (INOE-2000), Bucharest-Magurele, Str. Atomiștilor 409, RO-077125 Măgurele, Romania; qilib@yahoo.com

**Keywords:** cuprous oxide, thin film, magnetron sputtering, heterojunction, modelling, band alignment, interface defects

## Abstract

Silicon-based tandem solar cells incorporating low-cost, abundant, and non-toxic metal oxide materials can increase the conversion efficiency of silicon solar cells beyond their conventional limitations with obvious economic and environmental benefits. In this work, the electrical characteristics of a metal oxide thin-film heterojunction solar cell based on a cuprous oxide (Cu_2_O) absorber layer were investigated. Highly Al-doped n-type ZnO (AZO) and undoped p-type Cu_2_O thin films were prepared on quartz substrates by magnetron sputter deposition. The electrical and optical properties of these thin films were determined from Hall effect measurements and spectroscopic ellipsometry. After annealing the Cu_2_O film at 900 °C, the majority carrier (hole) mobility and the resistivity were measured at 50 cm^2^/V·s and 200 Ω·cm, respectively. Numerical modeling was carried out to investigate the effect of band alignment and interface defects on the electrical characteristics of the AZO/Cu_2_O heterojunction. The analysis suggests that the incorporation of a buffer layer can enhance the performance of the heterojunction solar cell as a result of reduced conduction band offset.

## 1. Introduction

The photovoltaic (PV) market is currently dominated by wafer-based crystalline silicon solar cells, with a market share of more than 90% [1]. Further cost reductions for this technology can be achieved by developing silicon-based tandem solar cells employing low-cost, abundant, and non-toxic metal oxide materials [2]. Among these metal oxides is cuprous oxide (Cu_2_O), which is considered an attractive material for photovoltaic applications since it is a p-type semiconductor with high optical absorption and a direct bandgap of about 2.1 eV, yielding a theoretical power conversion efficiency limit close to 20% under 1 sun illumination [3]. To construct a metal oxide p-n heterojunction, Cu_2_O can be combined with various n-type oxide materials, such as for example ZnO, and accordingly, one can foresee a heterojunction solar cell fully based on low-cost metal oxides. However, the highest conversion efficiency currently achieved experimentally for the n-ZnO/p-Cu_2_O heterojunction solar cell is only 8.1% [4], which suggests that further investigation of Cu_2_O-based solar cells is required in order to realize their full potential in photovoltaic applications.

For ZnO/Cu_2_O heterojunction solar cells, the properties of the heterojunction interface are critical in order to obtain high power conversion efficiency. For instance, reducing the defect density at the heterojunction interface to a minimum is important in order to avoid recombination losses. Unfortunately, the small enthalpy of formation for Cu_2_O (~170 kJ/mol) makes the interface prone to oxidation, e.g., a few nm thick intermediate CuO layer was reported to form at the ZnO/Cu_2_O interface [5], which may severely affect the current transport in Cu_2_O-based heterojunction devices since the defect density in this thin interface layer is typically much higher than that of the bulk Cu_2_O layer. Furthermore, the relatively low electron affinity of ~3.2 eV for Cu_2_O makes alignment of the energy bands challenging for ZnO/Cu_2_O heterojunction solar cells. For example, ZnO features a conduction and valence band misalignment with Cu_2_O of approximately 1 and 2.3 eV, respectively, causing a reduced open-circuit voltage (*V_oc_*) for the heterojunction solar cell [6]. To accommodate this band offset, it is possible to introduce a buffer layer between the ZnO and Cu_2_O layers in the heterojunction structure with the aim of improving the charge carrier transport properties of the heterojunction by reducing the band misalignment. Introduction of a buffer layer has shown to improve the power conversion efficiency of experimentally realized ZnO/Cu_2_O heterojunction solar cells in the last decade [7], e.g., for the 8.1% record cell utilization of a Zn_1−x_Ge_x_O buffer layer led to a significant improvement in device performance due to a reduction of the conduction band offset [4], but still, there is a rather large *V_oc_* deficit relative to the absorber bandgap suggesting that controlling the conduction band offset is essential in order to enhance the device performance [8]. Since the energy band alignment can be considerably affected by defects present at the heterojunction interface as well, it is important to understand the role of interface defects on the electrical characteristics of the heterojunction solar cell [9]. 

The objective of this work is to evaluate the electrical performance of a heterojunction solar cell based on Al-doped ZnO (AZO) and Cu_2_O thin films using numerical modelling. Device simulation is a prerequisite for designing efficient solar cells and for understanding the fundamental physical mechanisms, such as charge carrier transport and recombination. To this end, we have developed a device model for the AZO/Cu_2_O heterojunction using technology computer-aided design (TCAD) software, i.e., Silvaco Atlas. Material properties for sputter-deposited AZO and Cu_2_O thin films on quartz substrate were obtained from experimental characterization and implemented in the numerical model. Using this model, we investigate the effect of inserting a buffer layer with various electron affinity between the AZO and Cu_2_O layers on the electrical characteristics of the heterojunction solar cell. Moreover, the effect of the density of defects at the heterojunction interface as well as the density of bulk defects for the Cu_2_O absorber layer on the performance of the AZO/Cu_2_O heterojunction solar cell is analyzed. Current-voltage (I-V) parameters and energy band diagrams for the AZO/Cu_2_O heterojunction solar cells for different buffer layers and defect densities are presented and discussed. We show that the incorporation of a buffer layer can enhance the performance of the heterojunction solar cell as a result of reduced band offsets and that the energy band diagrams are affected by the defect density at the heterojunction interface.

## 2. Materials and Methods 

### 2.1. Thin Film Synthesis and Characterization

Cu_2_O and AZO thin films were deposited on 10 × 10 × 0.5 mm^3^ quartz substrates using a direct current/radio frequency (DC/RF) magnetron sputtering system (Semicore Triaxis). Then, 500 nm thick Cu_2_O films were deposited by reactive sputtering of a 99.999% Cu target in O_2_/Ar (6/49 sccm) at a substrate temperature of 400 °C. The power was fixed at 100 W. As-grown Cu_2_O films were annealed at 900 °C for 3 min in vacuum (pressure ~10^−1^ Torr). Then, 200 nm thick AZO films were deposited by co-sputtering of a 99.99% pure ZnO ceramic target at 50 W and a 99.999% Al target at 3 W in Ar at a substrate temperature of 400 °C, yielding an aluminum content of approximately 4 wt % in the deposited layers. During the magnetron sputtering deposition, the base pressure was below 4.0 × 10^−7^ Torr. The optical properties of the AZO and Cu_2_O thin films were analyzed using a Horiba Jobin Yvon Uvisel spectroscopic ellipsometer (Horiba Ltd., Kyoto, Japan). The optical transmittance spectrum was measured using a setup with spectrophotometers (Ocean Optics, Largo FL, USA), a deuterium–halogen light source, and an integrating sphere. Room temperature Hall effect measurements (LakeShore 7604) were carried out using the van-der Pauw configuration (Lake Shore Cryotronics, Inc., Westerville OH, USA).

### 2.2. Device Simulation

**Figure 1:**
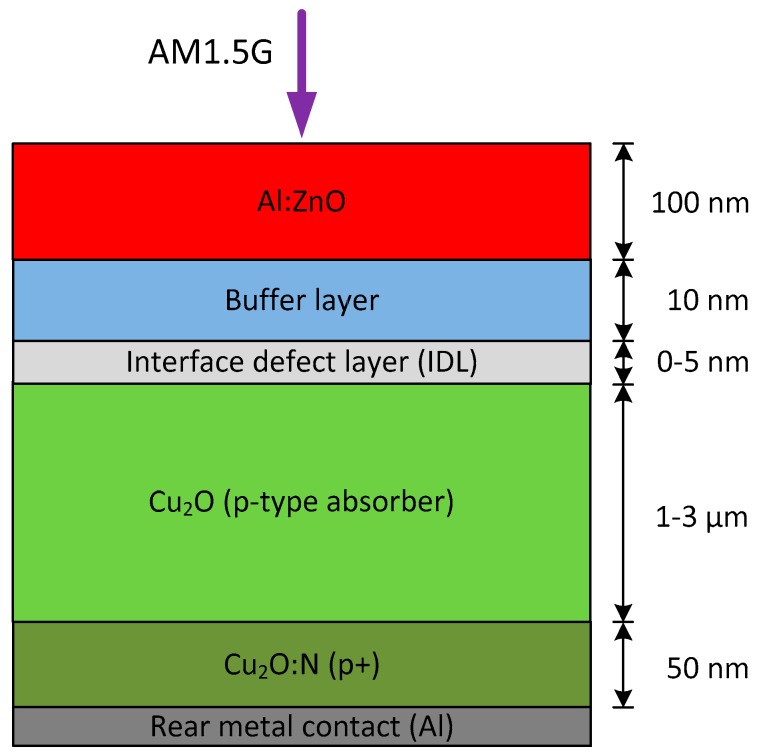
Schematic overview of the Al-doped ZnO (AZO)/Cu_2_O heterojunction model implemented in Silvaco Atlas.

The electrical performance of the AZO/Cu_2_O heterojunction solar cell with incorporation of different buffer layers was evaluated based on device modeling in Silvaco Atlas [10]. A schematic overview of the AZO/Cu2O heterojunction model is shown in Figure 1. The model assumes the heterojunction as several layers for which the physical properties were based on experimental and theoretical results reported in the literature [11]. Planar (flat) surfaces were adopted in the simulation model and the incident optical spectrum was air mass 1.5 global (AM1.5G). The thickness of the front side AZO layer was set to 100 nm in order to provide sufficient lateral transport of charge carriers for the solar cell. A buffer layer was inserted between the AZO transparent electrode and the Cu_2_O absorber layer. Additionally, an interface defect layer (IDL) was included between the buffer and Cu_2_O layers in order to represent interface defect states, which significantly influences the electrical characteristics of the AZO/Cu_2_O heterojunction. A highly doped Cu_2_O:N back surface layer was introduced at the rear side, forming a p+ back surface field in order to reduce the amount of recombination at the rear surface. The rear Al metal electrode was assumed to make an ohmic contact to the Cu_2_O:N back surface layer.

Table 1 summarizes the material parameters and corresponding values for each layer implemented in the AZO/Cu_2_O heterojunction model. Most of the parameter values were acquired from Ref. 11, except for the carrier mobilities and concentrations for the AZO and Cu_2_O layers which were experimentally derived from Hall effect measurements. The donor-like defects had a Gaussian distribution with a midgap level and a width of 0.1 eV was assumed for all layers [11]. The complex refractive indices for AZO and Cu_2_O were obtained by variable angle spectroscopic ellipsometry measurements. The reflectance of the incident light at the front surface was set to 5% whereas the internal reflectance at the rear surface was set to 90% for the entire wavelength range of the AM1.5G spectrum.

## 3. Results

### 3.1. Optical and Electrical Properties

**Figure 2:**
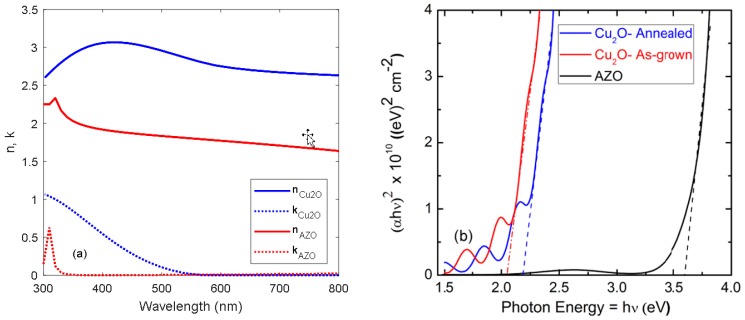
(**a**) Refractive index and extinction coefficient for AZO and annealed Cu_2_O thin films deposited on quartz; (**b**) Tauc plot for the as-grown and annealed Cu_2_O and AZO thin films deposited on quartz. The optical band gap values are estimated from extrapolation to the abscissa (dashed lines).

Figure 2a shows the complex refractive indices of the AZO and Cu_2_O thin films in the wavelength range from 300 to 800 nm, as obtained from spectroscopic ellipsometry measurements. The data suggests that high energy photons (λ < 550 nm) are absorbed in the Cu_2_O layer. The complex refractive indices for the AZO and Cu_2_O layers were implemented in the numerical model. Based on the measured transmittance spectra, a Tauc plot analysis was carried out in order to determine the optical band gap of the AZO and Cu_2_O (as-grown and annealed) thin films [12]. 

Figure 2b shows the resulting Tauc plots, which suggest that the optical band gap is increased from *E*_g_ = 2.06 eV for the as-grown Cu_2_O film to *E*_g_ = 2.19 eV after annealing at 900 °C. The widening of the optical band gap after annealing might be due to partial elimination of defects states [13] and more phase-pure Cu_2_O films with negligible contribution of the CuO phase. For the AZO thin film, the optical band gap was estimated to *E*_g_ = 3.61 eV, evidencing the so-called Burstein–Moss effect. 

The majority carrier mobility and concentration, as well as the film resistivity for AZO and (as-grown and annealed) Cu_2_O thin films deposited on quartz are summarized in Table 2. The data suggests that the electrical properties for the Cu_2_O thin film are enhanced after thermal annealing at 900 °C, i.e., the resistivity decreases from 560 to 200 Ω·cm and the majority carrier (hole) mobility increases from 10 to 50 cm^2^/V·s after annealing. These values are comparable to those reported previously for sputter-deposited polycrystalline Cu_2_O thin films on quartz [14,15], suggesting that the annealed Cu_2_O thin films are well suited for photovoltaic applications. The increase in carrier mobility after annealing can, at least partly, be attributed to the increase in grain size and reduced grain-boundary scattering [16,17].

### 3.2. Modelling of AZO/Cu_2_O Heterojunction

**Figure 3:**
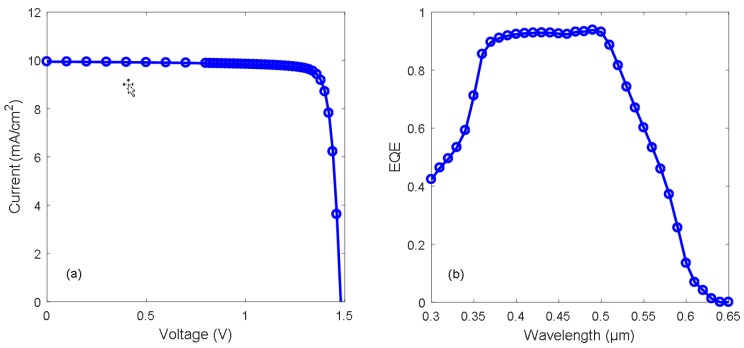
Simulated (**a**) I–V and (**b**) external quantum efficiency (EQE) curve for the AZO/Cu_2_O heterojunction solar cell under 1 sun illumination, using the default parameter values listed in Table 1.

Figure 3a shows the simulated I–V curve for the AZO/Cu_2_O heterojunction solar cell under 1 sun (AM1.5G) illumination. In this case, the default parameter values provided in Table 1 for the different layers constituting the heterojunction structure were implemented, yielding a short circuit current density (*J*_sc_) of 10.0 mA/cm^2^, an open circuit voltage (*V*_oc_) of 1.48 V, a fill factor (FF) of 84.7%, and a power conversion efficiency (*η*) of 12.5%. Figure 3b shows the corresponding simulated external quantum efficiency (EQE) curve for the AZO/Cu_2_O heterojunction solar cell. The EQE is approximately 90% in the wavelength range from 400 to 500 nm, in good agreement with the simulated EQE curve reported previously for a corresponding AZO/Cu_2_O heterojunction solar cell model [11].

#### 3.2.1. Effect of Buffer Layer Electron Affinity

**Figure 4:**
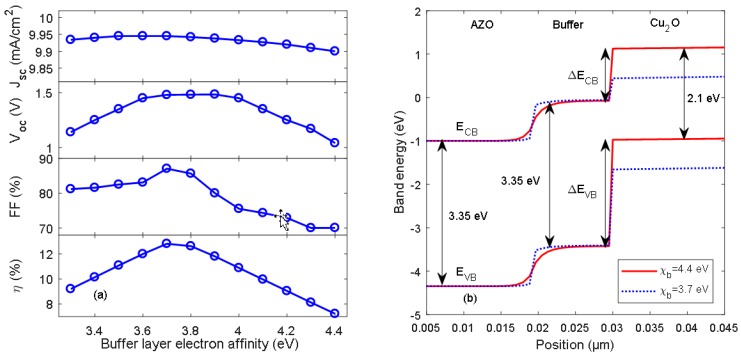
(**a**) Simulated I–V parameters for the AZO/Cu_2_O heterojunction solar cell as a function of the buffer layer electron affinity. (**b**) Energy band diagram for the AZO/Cu_2_O heterojunction interface for a buffer layer electron affinity (χ_b_) of 3.7 and 4.4 eV.

Figure 4a shows the simulated I–V parameters for the AZO/Cu_2_O heterojunction solar cell as a function of the buffer layer electron affinity, without an interface defect layer included in the structure. For the heterojunction with a buffer layer electron affinity χ_b_ = 4.4 eV, corresponding to that of AZO, a conversion efficiency of 7.2% was achieved. By introducing a buffer layer with lower electron affinity, the electrical performance was significantly improved, and the highest performance (*η* = 12.5%) was achieved for a buffer layer electron affinity χ_b_ = 3.7 eV. The corresponding energy band diagram for the AZO/Cu_2_O heterojunction interface is shown in Figure 4b, for a buffer layer electron affinity of 3.7 and 4.4 eV. *E*_CB_ and *E*_VB_ correspond to the conduction band minimum energy and valence band maximum energy, respectively. In addition, indicated in Figure 4b are the band gap energy offset for the conduction band (∆*E*_CB_) and valence band (∆*E*_VB_) along with the energy band gaps, *E*_g_. The band diagram suggests that ∆*E*_CB_ and ∆*E*_VB_ are reduced with the introduction of a buffer layer, i.e., ∆*E*_CB_ is reduced from 1.05 to 0.38 eV and ∆*E*_VB_ is reduced from 2.45 to 1.75 eV when a buffer layer with electron affinity χ_b_ = 3.7 eV is introduced at the heterojunction interface. As will be discussed in the next section, various thin film materials with electron affinity lower than that of ZnO (χ_ZnO_ = 4.4 eV) have been implemented as buffer layer in experimentally realized ZnO/Cu_2_O heterojunction solar cells in order to enhance their electrical performance [6,7,8,9].

#### 3.2.2. Effect of Interface Defects

**Figure 5:**
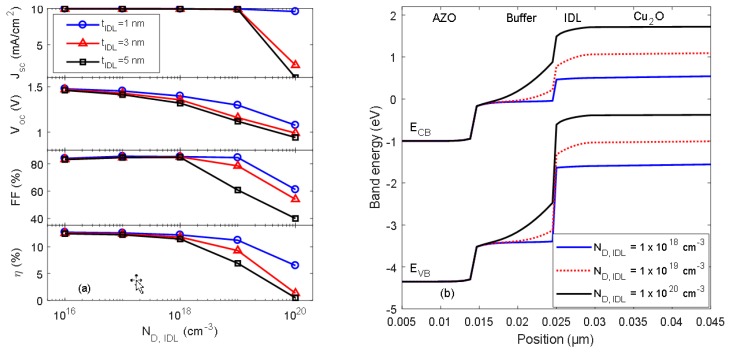
(**a**) Simulated I–V parameters for the AZO/Cu_2_O heterojunction solar cell as a function of *N*_D,IDL_ for various interface defect layer thicknesses (*t*_IDL_); (**b**) energy band diagram for the AZO/Cu_2_O heterojunction interface for various *N*_D, IDL_ with *t*_IDL_ = 5 nm.

Figure 5a shows the simulated I–V parameters for the AZO/Cu_2_O heterojunction solar cell as a function of interface defect density (*N*_D,IDL_) for different thicknesses of the interface defect layer (*t*_IDL_). The defect density and layer thickness were varied in the ranges 1 × 10^16^ to 1 × 10^20^ cm^−3^ and 1–5 nm, respectively. The results suggest that the electrical performance is severely affected for an interface defect density *N*_D,IDL_ >1 × 10^18^ cm^−3^, i.e., for *t*_IDL_ = 5 nm the conversion efficiency decreases from *η* = 11.5% for *N*_D,IDL_ = 1 × 10^18^ cm^−3^ to *η* = 0.5% for *N*_D,IDL_ >10^20^ cm^−3^. Figure 5b shows the corresponding energy band diagram for the AZO/Cu_2_O heterojunction interface for *t*_IDL_ = 5 nm and different *N*_D,IDL_. The magnitude of band bending in the buffer layer and interface defect layer at the heterojunction interface increases with *N*_D,IDL_. As a result, the energy bands for the Cu_2_O absorber layer are shifted towards higher energies.

#### 3.2.3. Effect of Cu_2_O Bulk Defects

**Figure 6:**
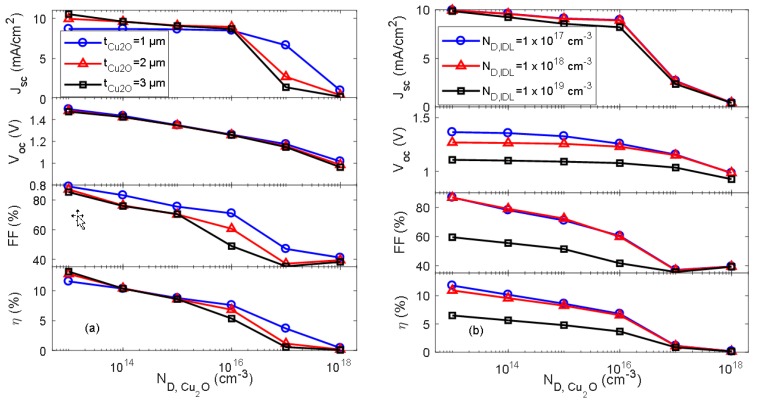
Simulated I–V parameters for the AZO/Cu_2_O heterojunction solar cell as a function of *N*_D,Cu2O_ for (**a**) various interface absorber layer thicknesses (*t*_Cu2O_) and (**b**) various defect densities in the interface defect layer (IDL) (*N*_D, IDL_) for *t*_IDL_ = 5 nm.

Figure 6a shows the simulated I–V parameters for the AZO/Cu_2_O heterojunction solar cell as a function of Cu_2_O bulk defect density (*N*_D, Cu2O_) for different thicknesses of the Cu_2_O absorber layer (*t*_Cu2O_). *N*_D, Cu2O_, was varied in the range 1 × 10^13^ to 1 × 10^18^ cm^−3^. The results suggest that the electrical performance of the AZO/Cu_2_O heterojunction solar cell is reduced with increasing *N*_D, Cu2O_, e.g., for *t*_Cu2O_ = 2 µm the conversion efficiency decreases from 10.4% for *N*_D, Cu2O_ = 1 × 10^14^ cm^−3^ to 1.1% for *N*_D, Cu2O_ = 1 × 10^17^ cm^−3^. Figure 6b shows the simulated I–V parameters for the AZO/Cu_2_O heterojunction as a function of *N*_D, Cu2O_ for different IDL defect densities. The thickness of the interface defect layer was set to 5 nm in this simulation. 

## 4. Discussion on Heterojunction Interface Properties and Defect Analysis

The numerical analysis shows that the buffer layer electron affinity has a big impact on the electrical performance of the ZnO/Cu_2_O heterojunction solar cell. Figure 4 displays a distinct improvement in *V*_oc_ and FF as the buffer layer electron affinity is reduced from χ_b_ = 4.4 eV to χ_b_ = 3.7 eV, as a result of reduced conduction and valence band offsets giving a corresponding reduction of charge carrier recombination at the heterojunction interface. Additionally, the charge carrier collection is improved as a result of the suppressed interface recombination [11]. For χ_b_ = 3.7 eV, a cliff-type conduction band offset ∆*E*_CB_ = 0.38 eV is observed. Previous studies of the ZnO/Cu_2_O heterojunction have indicated that a conduction band offset of 0.2–0.3 eV yields the highest open circuit voltage [8,9]. To lower the conduction band offset for the ZnO/Cu_2_O heterojunction solar cell there are several materials that can potentially be used as buffer layers, including Ga_2_O_3_ (χ = 4.0 eV), TiO_2_ (χ = 4.0 eV), ZnS (χ = 3.9 eV), GaN (χ = 3.5 eV), Ta_2_O_5_ (3.2 eV), and various ZnO alloys [11,18,19]. Many of these materials have been implemented as buffer layer in experimentally realized ZnO/Cu_2_O heterojunction solar cells, but a relatively low performance has usually been achieved [7,20]. This suggests that manipulation of the energy band alignment at the heterojunction interface is most likely required, for instance by adjusting the deposition conditions, in addition to the incorporation of a buffer layer, in order to develop a high performance ZnO/Cu_2_O heterojunction solar cell [9]. 

The energy band diagrams presented in Figure 5b suggest that the defect density at the heterojunction interface affects the amount of band bending in the IDL and buffer layer, and the electrical performance of the ZnO/Cu_2_O heterojunction solar cell is drastically reduced for an interface defect density *N*_D,IDL_ > 10^18^ cm^−3^, as shown in Figure 5a. The concentration and charge transition energies of defects add restrictions to the position of the Fermi energy level in the materials, which may influence the energy band alignment at the heterojunction interface [9]. Thus, the alignment of the energy bands and the charge carrier transport and recombination at the heterojunction interface are dependent not only on the buffer layer affinity but also on electronic properties of the interfacial region of the Cu_2_O layer. The small enthalpy of formation for Cu_2_O means that Cu and CuO may easily be formed when the buffer layer is deposited onto the Cu_2_O surface. Typically, interfacial Cu will form a low barrier Schottky diode with Cu_2_O, lowering the open circuit voltage of the heterojunction solar cell, whereas interfacial CuO could result in Fermi level pinning due to the low band gap (*E*_g_ = 1.2 eV) of CuO [21]. It was previously found that the presence of CuO at the interface causes a 0.4 eV shift in the valence-band offset between Cu_2_O and ZnO, which may explain the large variation in the ZnO/Cu_2_O valence-band offset reported in the literature [9,21]. Additionally, it was demonstrated a significantly higher *V*_OC_ for a ZnO/Cu_2_O heterojunction solar cell with a stoichiometric (Cu_2_O) interface compared to that achieved with a mixed phase interface [21]. Thus, it is critical to be able to tune and control the oxidation state of copper oxide between CuO, Cu_2_O, and Cu at the ZnO/Cu_2_O interface by carefully adjusting the deposition conditions during deposition of the buffer layer. 

Figure 6 suggests that the performance of the AZO/Cu_2_O heterojunction solar cell is significantly reduced with increasing bulk defect density in the Cu_2_O layer. The increased carrier recombination in the bulk of the absorber layer and at the heterojunction interface affects both the short circuit current and the open circuit voltage detrimentally, e.g., *J*_sc_ is reduced from approximately 9 mA/cm^2^ for *N*_D, Cu2O_ = 1 × 10^16^ cm^−3^ to less than 1 mA/cm^2^ for *N*_D, Cu2O_ = 1 × 10^18^ cm^−3^. This implies that controlling the bulk defect density during the thin film processing could also play an important role for the performance of the ZnO/Cu_2_O heterojunction solar cell. The phase-purity and stoichiometry for the Cu_2_O absorber layer would ideally be controlled during the deposition process, but may also be modified by post-deposition processing such as thermal annealing or ion-implantation of for instance hydrogen or nitrogen. For example, passivation of donor-like defects has been reported by exposing Cu_2_O films to hydrogen plasma [22]. Experimental investigation of the ZnO/Cu_2_O heterojunction has shown that the conduction and valence band offsets can indeed be altered by intrinsic defects and impurities, which suggests that controlling the interface formation during the fabrication process is critical to the device performance and functionality [9]. Such band alignment engineering of the metal oxide heterojunction, where the functional behavior can be tailored by the way the bands align at the interface, could potentially provide a pathway to realization of high-efficiency ZnO/Cu_2_O heterojunction solar cells.

## 5. Conclusions

In summary, the electrical characteristics of a metal oxide thin-film heterojunction solar cell based on a Cu_2_O absorber layer were investigated through numerical simulation using Silvaco Atlas. The electrical and optical properties of AZO and Cu_2_O thin films prepared on quartz substrates by magnetron sputter deposition were determined from spectroscopic ellipsometry and Hall effect measurements and implemented in the device model. The influence of the electronic properties of the heterojunction interface on the performance of an AZO/Cu_2_O heterojunction solar cell was analyzed, and the results suggest that a conversion efficiency of more than 12% can be achieved when a buffer layer with electron affinity χ_b_ = 3.7 eV is inserted between the AZO transparent electrode and the Cu_2_O absorber layer. Thus, the sputter-deposited Cu_2_O thin films show good promise as absorber layer for photovoltaic applications. In order to develop a high performance Cu_2_O-based heterojunction solar cell with a sufficiently low conduction band offset, manipulation of the energy band alignment at the heterojunction interface will most likely be required, for instance by adjusting the deposition conditions or by post-deposition treatment of the Cu_2_O layer, in addition to incorporation of a buffer layer. One of the main advantages of the magnetron sputtering technique, which has been used to synthesize the metal oxide thin films in this work, is that it provides good control of the stoichiometry of the films by varying the process parameters, such as substrate temperature, gas mass flows, and target power density, e.g., in order to obtain Cu-rich or O-rich phases for Cu_2_O [12,16]. This aspect, combined with the ability to control the electrical properties of the sputter-deposited thin films by rapid thermal annealing [17], suggests that it can indeed be possible to modify the band alignment at the interface to improve the electrical characteristics of the AZO/Cu_2_O heterojunction. The impact of hydrogen ion-implantation and subsequent thermal annealing of the Cu_2_O layer on the electrical properties of the AZO/Cu_2_O heterojunction will be the subject of further investigation. Additionally, further development of the numerical model to account for excitonic effects will be looked into. The traditional free carrier model has been reported not to accurately capture the fundamental photoconversion and charge-transport mechanism in Cu_2_O, resulting in a significant underestimation of the conversion efficiency of Cu_2_O-based heterojunction solar cells [23].

## Figures and Tables

**Table 1:** Material parameters and corresponding default values used for simulation of the AZO/Cu_2_O heterojunction in Silvaco Atlas. The asterisk (*) indicates values that were varied in the numerical analysis.

Parameter	AZO	Buffer Layer	IDL	Cu_2_O	Cu_2_O:N
Layer thickness (nm)	100	10	0 *	2000 *	50
Electron affinity (eV)	4.4	3.7 *	3.2	3.2	3.2
Band gap (eV)	3.35	3.35	2.1	2.1	2.1
Relative permittivity	9	9	7.6	7.6	7.6
Acceptor concentration (cm^−3^)	0	0	1 × 10^15^	1 × 10^15^	1 × 10^21^
Donor concentration (cm^−3^)	4 × 10^20^	1 × 10^19^	0	0	0
Hole mobility (cm^2^/V·s)	5	5	50	50	50
Electron mobility (cm^2^/V·s)	10	10	100	100	100
Effective density of states in conduction band (cm^−3^)	2.2 × 10^18^	2.2 × 10^18^	2.4 × 10^19^	2.4 × 10^19^	2.4 × 10^19^
Effective density of states in valence band (cm^−3^)	1.8 × 10^19^	1.8 × 10^19^	1.3 × 10^19^	1.3 × 10^19^	1.3 × 10^19^
Capture cross section of holes (cm^2^)	1 × 10^−15^	1 × 10^−15^	1 × 10^−13^	1 × 10^−15^	1 × 10^−15^
Capture cross section of electrons (cm^2^)	1 × 10^−12^	1 × 10^−12^	1 × 10^−13^	5 × 10^−13^	5 × 10^−13^
Defect density (donor-like) (cm^−3^)	1 × 10^18^	5 × 10^17^	1 × 10^19^ *	1 × 10^13^ *	1 × 10^13^
Defect peak energy (eV)	1.68	1.68	1.05	1.05	1.05
Defect distribution width (eV)	0.1	0.1	0.1	0.1	0.1

**Table 2:** Majority carrier mobility and concentration, as well as film resistivity for AZO and Cu_2_O thin films on quartz, derived from room temperature Hall effect measurements.

Parameter	AZO	Cu_2_O	Cu_2_O *
Mobility (cm^2^/V·s)	20	10	50
Concentration (cm^-3^)	3 × 10^20^	3 × 10^15^	1 × 10^15^
Resistivity (Ohm·cm)	5 × 10^−4^	560	200

* Annealed at 900 °C.
